# Hardware Accelerated Compression of LIDAR Data Using FPGA Devices

**DOI:** 10.3390/s130506405

**Published:** 2013-05-14

**Authors:** Anton Biasizzo, Franc Novak

**Affiliations:** Jozef Stefan Institute, Jamova 39, Ljubljana 1000, Slovenia; E-Mail: anton.biasizzo@ijs.si

**Keywords:** data compression, LIDAR, FPGA, hardware acceleration

## Abstract

Airborne Light Detection and Ranging (LIDAR) has become a mainstream technology for terrain data acquisition and mapping. High sampling density of LIDAR enables the acquisition of high details of the terrain, but on the other hand, it results in a vast amount of gathered data, which requires huge storage space as well as substantial processing effort. The data are usually stored in the LAS format which has become the *de facto* standard for LIDAR data storage and exchange. In the paper, a hardware accelerated compression of LIDAR data is presented. The compression and decompression of LIDAR data is performed by a dedicated FPGA-based circuit and interfaced to the computer via a PCI-E general bus. The hardware compressor consists of three modules: LIDAR data predictor, variable length coder, and arithmetic coder. Hardware compression is considerably faster than software compression, while it also alleviates the processor load.

## 1. Introduction

Light Detection and Ranging (LIDAR) is an optical remote sensing technique that measures the distance to the surface of a distant object. Like radar technology, the distance is determined by illuminating the target by a light source, typically a laser, and measuring the time between the light emission and the detection of the reflection. Since LIDAR uses electromagnetic radiation with much shorter wavelength then radar, it can detect much smaller objects and has finer resolution [1].

LIDAR data scanners used for collecting terrain data are usually mounted on airplanes. Shooting over 100,000 laser pulses per second to the terrain surface, the measurement resolution exceeds one point per square meter. This way, a huge amount of high precision spatial data [2–6] of cities [7,8], or countries [9] can be collected. In order to determine the coordinates of sampled points the position and the orientation of the LIDAR scanner must be known. Thus the airplanes are also equipped with global positioning system (GPS) equipment to determine their position and inertial measurement units (IMUs) to determine the orientation of the airplane and the LIDAR scanner [10].

The scanner monitors the reflection signal for each laser pulse that was sent out. The intensity peaks of the signal correspond to the objects that were hit by the laser beam and reflected a portion of the beam back to the scanner. The notable width of the laser beam can cause partial reflections on several objects such as branches, leaves, birds, wires, before reaching the ground, which results in several intensity peaks for a single shot. Each peak that exceeds certain threshold is called a return. The coordinates of the objects, that caused the returns, together with the intensity, GPS time, return number, etc. are recorded by the scanner. They are needed for subsequent off-line processing.

LIDAR scanners usually store the acquired data in a binary, vendor specific format. These data formats are proprietary, vary from vendor to vendor, and thus they are not suitable for data processing and exchange. To achieve interoperability between different LIDAR vendors and users, the American Society for Photogrammetry and Remote Sensing (ASPRS) developed a simple binary exchange file format known as the LAS format [11].

Due to the scanner high resolution, the amount of collected LIDAR data is vast and may amount to millions or even billions of points. This presents a serious problem for data exchange, distribution, and even for the storage of data. Data compression may remedy these problems. In general, data compression can be either lossless, where the original data set can be fully restored during decompression, or lossy, where some information is discarded during the compression. Lossy data compression can achieve better compression ratio and is typically used for data visualization where a lot of details may be omitted. However, for the processing and distribution of the LIDAR data, lossless methods are required. Furthermore, the LIDAR data has to be compressed without any modification, which means that reordering of points is not permitted.

Data compression efficiently reduces the problems of storing and distribution of LIDAR data but it also introduces additional load to the processing unit and consequently increases processing time. In order to tackle the problem we developed a FPGA based hardware LIDAR compressor. The compressor is based on the LAScompression algorithm [12], which is described in more detail in a subsequent section. The developed hardware compressor consists of predictor modules for the LIDAR point attributes, local memory used for storing intermediate data, the variable length coder, and the arithmetic coder. The LASCompression algorithm was adjusted in order to achieve better hardware implementation while preserving the exact values of the intermediate and final data. Each building module processes an attribute during single clock period, which yields a maximal data throughput with a given clock source. The hardware compressor also exploits parallelisms in the method and the compression is 250 times faster than the software variant. Furthermore, while the data are compressed, the processor can perform some other tasks since no processor interaction is needed. To the best of our knowledge, no such hardware implementation has been reported by the time of writing this paper.

In the following, data compression techniques are briefly reviewed. In Section 3 a brief overview of the LAS data format is given and in Section 4 the LAScompression algorithm is described. In Section 5 the proposed hardware implementation is described. The results are presented in Section 6, and Section 7 concludes the paper.

## 2. Data Compression Techniques

General purpose compression schemes such as ZIP or RAR are lossless and could be applied for LIDAR data compression. They are, however, not very effective, because they do not have any insight into the LIDAR data structure to accurately model the probabilities of data patterns. Dedicated schemes for compression of geometric data would be expected to give better compression ratios as well as better performance, yet they have drawbacks which prevent their application in the case of LIDAR data compression. For example, compression methods developed for triangular meshes such as those described in References [13–16] reorder triangular meshes to achieve maximal compression. They are not directly applicable for LIDAR compression since in this case data reordering is not permitted. Likewise, some (general purpose) point-cloud compression methods, such as Reference [17], also rely on point reordering to achieve high compression ratios. On the other hand, the point-cloud compression methods using spatial subdivision, like Reference [18] do not generalize well for other point attributes like color and GPS time. Point-cloud compression method based on fitting points in curves is described in Reference [19], however other point attributes like color and GPS time are not addressed and according to the authors the integration of other point attributes remains subject of further research.

LAScompression [20], LASzip [21], and LizardTech^®^ LiDAR compressor™ [22] are all point-cloud compression methods that were developed to lossless compress LIDAR points stored in the LAS format. LAScompression [20] and LASzip [21] compress blocks of LIDAR points by prediction coding. They both predict the attributes of a new point from the set of previous points using predictors. The corrective deltas are then compressed with arithmetic coding [23,24]. LizardTech's LiDAR compressor [22] compresses blocks of LIDAR points by a simplified Haar wavelet transformation on each point attribute individually. The coefficients are then compressed by arithmetic coding. The GPS time attribute is compressed by the standard deflate method. LAScompression and LASzip are publically available while LizardTech^®^ LiDAR compressor™ is commercial.

As regards hardware implementations, no LIDAR data compressor has yet been reported. There are some off-the-shelf hardware compressors on the market that implement general purpose compression algorithms but such compression methods are not efficient for LIDAR data compression. For the hardware implementation in our case, the LAScompression algorithm was selected because of its high efficiency and the availability of algorithm details.

## 3. LAS Format

The LAS binary exchange format [11] has become the *de facto* industry standard for storing and exchange of LIDAR data. During the last years several new features were added to the LAS format, however all versions share three main sections:

- A header, which contains general information of the LIDAR data, such as version of the LAS format, number of acquired points, scaling factor for the coordinates, *etc.*,
- Variable-length records with additional information such as projection, meta-data, user data,
- A set of fixed-length point data records, where the coordinates *x*, *y*, *z* and other point attributes like GPS time, point type, *etc.*, are stored.

**Figure 1.**
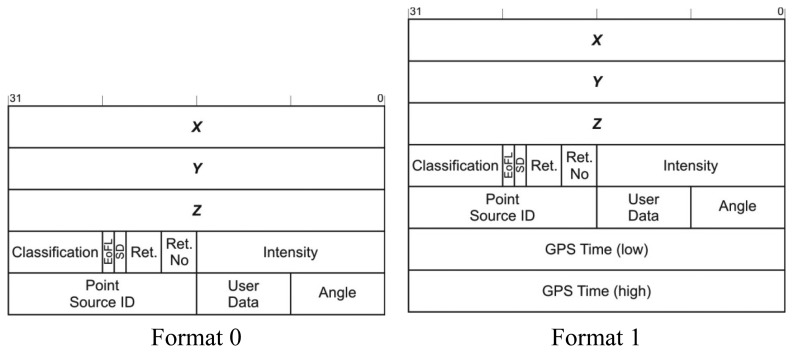
LIDAR point data record structures defined by LAS format version 1.1.

The header of the LAS file as well as a variable length additional information describe the LIDAR data file as a whole and represent only a small fraction of the LAS file. Therefore it is the set of point data records that are targeted by the compression method. The LAS format version 1.1 defines two different point data record formats shown in Figure 1.

Linear transformation of the coordinates is used in the LAS format to achieve specified precision and the scaling factors and offsets are stored in the header. This way the data producer can select the representation quantization while taking into account the actual acquisition precision. In order to reduce the noise, *x*, *y*, and *z* coordinates are stored as signed integers.

## 4. LAScompression Algorithm

**Figure 2.**
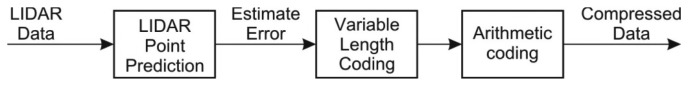
The structure of LAScompression algorithm.

In LIDAR data acquisition, points are acquired and recorded in series by moving and tilting the laser gun. The LAScompression algorithm [12] exploits the correlation of the points implied by the movement of the laser beam. Each point stored in the LAS format is a record of attributes consisting of *x*, *y*, *z* coordinates and the associated attributes like intensity, return number, scan direction flag, edge of a flight line, classification index, scan angle rank, GPS time, *etc.* The compression algorithm splits attributes into different streams which are compressed independently. The compression of each stream is performed by three consecutive independent steps as depicted in Figure 2:

- Predictive coding, where the difference between the point attribute estimations and the actual values is determined,
- Variable length encoding (VLC) of the obtained error values,
- Entropy based compression of results of VLC encoding by arithmetic coding [23,24].

### 4.1. Predictive Coding

Predictive coding is an efficient compression technique when the data stream is correlated. The efficiency of the predictive coder depends on the quality of the predictor. LIDAR data are recorded and stored in order with respect to the laser beam movement. Consequently, the estimation of a future point can be determined from the previous points. In general, all previous points could be used in the estimation of the future point however it is obvious that only the recent points have a significant correlation with the future point. Hence in the prediction of a future point only a few recent points are used.

Let *p_i_* be the *i*-th point in the sequence and let *p*'*_i_* be the estimated point determined by the prediction function *f(P)*, where *P* contains the set of the last previous points. The result of the predictive coder is the error *e_i_* which represents the difference between the actual value and the prediction given by Equation (1):

(1) $$\begin{array}{l} e_{i}=p_{i}-p_{i}^{\prime }\\ p_{i}^{\prime }=f\;(P)=f\;(p_{i-1},p_{i-2}\;,\ldots ,p_{0}) \end{array}$$

As mentioned before, the LAScompression method splits LAS point attributes into different streams hence there are different predictors for each stream. LAS compression method uses four different predictors [12]: X coordinate predictor, Y coordinate predictor, Z coordinate predictor and linear predictor.

#### 4.1.1. *X*-Coordinate Prediction

When acquiring LIDAR data, we distinguish between two different cases regarding the way the terrain points are generated. Two successive points originate

- From the reflections of two successive laser bursts from the same plane, usually from the ground, or
- From the reflections of a single laser burst from different planes *i.e.*, from tree leaves and the ground (multiple reflections).

When two successive points correspond to the reflections of successive laser bursts on the same plane, the distance of both points is relatively small. Furthermore, since the LIDAR resolution is very high, the distance is almost invariant in such cases. On the other hand, when the successive points correspond to multiple reflections, the point distances could differ significantly, which in turn results in larger distance between the points. Such a distance cannot be predicted hence only the first option is covered by the predictor. The points corresponding to the reflections from other planes would mislead the prediction of the next point. Consequently the points which vary significantly from the current set of points are ignored.

The estimation of the next coordinate *x*^′^*_i_* is given as a displacement of the last coordinate *x_i-1_* by an average distance of *k_x_* previous distances between successive points. The extreme values are discarded from the set of distances in order to eliminate the distances from the same laser burst. The predictor for the *x* coordinate is given by Equation (2):

(2) $$\begin{array}{cc} x_{i}^{'} & =x_{i-1}+\bar{\Delta x_{i}}\\ \bar{\Delta x_{i}} & =\frac{\sum_{j=i-1}^{i-k_{x}}\;x_{j}-x_{j-1}}{k_{x}} \end{array}$$

From the experimental studies it was concluded that it is sufficient if the number of previous distances *k_x_* is set to 8.

#### 4.1.2. *Y*-Coordinate Prediction

The correlations presented for the prediction of *x* coordinate are also valid for the prediction of *y* coordinate. However the *y* coordinate prediction can cover both scenarios since *x* coordinate is already known and can be used in the prediction. In the case of multiple reflections, the laser beam strikes the surface at a certain angle. In this case the difference *Δx* can be used to predict *y* coordinate. When the successive points are not the result of a single laser burst, other parameters like flight direction and scan angle affect the point positions. In order to achieve accurate prediction, two prediction rules are used:

- If a similar *Δx* is found among the last *k_y_* successive points ***p*** then its *Δy* is used as an estimation of the current difference of *y* coordinate,
- If there are no such differences, a linear prediction based on Δ*x* is used.

The predictor for y coordinate is given by Equation (3):

(3) $$\begin{array}{ll} y_{i}^{'} & =y_{i-1}+\left(\begin{array}{ccc} \Delta y_{n} & ; & \Delta x_{n}≅\Delta x_{i},i-k_{y}\leq n<i\\ \Delta_{xi}\cdot k_{\Delta x} & ; & \text{else} \end{array}\right),\\ \Delta x_{i} & \begin{array}{cc} =x_{i}-x_{i-1}, & \Delta y_{i}=y_{i}-y_{i-1} \end{array}\\ \Delta_{xi} & \begin{array}{cc} =\Delta x_{i}-\Delta x_{i-1}, & k_{\Delta x}=\frac{\Delta y_{i-1}-\Delta y_{i-2}}{\Delta x_{i-1}\;-\Delta x_{i-2}} \end{array} \end{array}$$

Like in the case of predictor for *x* coordinate, the parameter *k_y_* was determined through experiments and set to 100.

#### 4.1.3. *Z*-Coordinate Prediction

The same scheme used in the prediction of the *y* coordinate, applies for the prediction of the *z* coordinate. However, for the prediction of the *z* coordinate either difference *Δx* or *Δy* can be used. To get better predictions three prediction rules are used:

- If there are two successive points with similar *Δx* and *Δy* among the last *k_z_* samples, then the associated *Δz* is used as an estimation of the current difference of *z* coordinate,
- If the previous predictions based on *Δx* are better, than the predictions based on *Δy* then linear predictions based on *Δy* are used,
- Otherwise, linear prediction based on *Δy* is used.

In order to select linear prediction rule based on *Δx* or *Δy*, two statistical variables *errorX* and *errorY* that measure previous prediction accuracy are employed.

The predictor for the *y* coordinate is given by Equation (4):

(4) $$\begin{array}{l} z_{i}^{'}=z_{i-1}+\left(\begin{array}{ccc} \Delta z_{n} & ; & \left(\Delta x_{n},\Delta y_{n}\right)≅\left(\Delta x_{i},\Delta y_{i}\right),i-k_{z}\leq n<i\\ \Delta_{xi}\cdot k_{\Delta x} & ; & \text{errorX}\leq \text{errorY}\\ \Delta_{yi}\cdot k_{\Delta y} & ; & \text{errorX}>\text{errorY} \end{array}\right),\\ \begin{array}{lll} \begin{array}{ll} \Delta x_{i} & =x_{i}-x_{i-1},\\ \Delta_{xi} & =\Delta x_{i}-\Delta x_{i-1},\\ k_{\Delta x} & =\frac{\Delta z_{i-1}-\Delta z_{i-2}}{\Delta x_{i-1}-\Delta x_{i-2}}, \end{array} & \begin{array}{ll} \Delta y_{i} & =y_{i}-y_{i-1}.\\ \Delta_{yi} & =\Delta y_{i}-\Delta y_{i-1,}\\ k_{\Delta y} & =\frac{\Delta z_{i-1}-\Delta z_{i-2}}{\Delta y_{i-1}-\Delta y_{i-2}} \end{array} & \Delta z_{i}=z_{i}-z_{i-1} \end{array} \end{array}$$

Like in the case of the *x*-coordinate predictor and *y*-coordinate predictor, the parameter *k_z_* was determined through experiments and set to 100.

#### 4.1.4. Prediction of Other Attributes

Other attributes like source ID and scan angle rarely change. When they do, they change by a constant amount, so for them, a linear predictor can be used. The last difference of the attribute is used as the displacement from the previous value. The predictor for such attributes is given by Equation (5):

(5) $$\begin{array}{cc} s_{i}^{'} & =s_{i-1}+\Delta s_{i-1},\\ \Delta s_{i} & =s_{i}-s_{i-1} \end{array}$$

The result of the predictive coding are streams of prediction errors for each LAS format attribute and are fed into variable-length coder. From the description of the coordinate predictors it is obvious that the *x* coordinate has to be decoded prior to decoding the *y* coordinate and both the *x* and *y* coordinates have to be decoded prior to decoding the *z* coordinate.

### 4.2. Variable-Length Coding

The presented prediction coding significantly reduces the absolute values of the attributes. While the absolute values in the data stream are reduced, the size of the stream remains intact. Thus the resulting stream has a lot of null values which can be omitted using variable length encoding.

The attributes in LAS format are signed integer values stored in at most 4 bytes. The variable-length encoding of an input stream is performed in the following steps:

- The input stream is split into two streams: the description stream and the absolute value stream.
  ○ With each value *s* in the input stream a description *d* is associated. The description *d* holds the information of the sign of the input value and the length of the minimum byte representation of the value *s*. Since integers are stored in 4 bytes there are at most 9 different values of description *d* and it can be stored using only 4 bits. The whole description is stored in a byte where the non-used bits are set to 0. The description bytes *d* form a new description stream.
  ○ For each value *s* in the input stream an absolute value *|s|* is determined.
- The leading zero bytes in the absolute value stream are removed and the stream is further decomposed into four byte streams with respect to byte significance.

By this procedure the variable length encoding splits the stream of integer values into five streams: the description stream and four data byte streams. These streams are then individually fed to the arithmetic coder in the predefined order: the description stream is fed first followed by the other streams with respect of their byte significance.

### 4.3. Arithmetic Coding

Arithmetic coding is an entropy coding method that is superior to the well-known Huffman coding and achieves near entropy limit compression ratios for a long data sequence [23,24]. In its general form arithmetic coding algorithm allows any input alphabet, including binary and byte alphabets. The binary arithmetic coding is an attractive choice for compression of binary streams since other compression algorithms like Huffman coding do not work at all, but a multi-symbol alphabet like a byte alphabet gives even better compression results.

The arithmetic coding allows separation of input symbol model and compression algorithm which in turn allows more sophisticated model implementation like high-order models and adaptive models. While these model implementations may increase the compression efficiency they also increase the computational complexity. The input of the arithmetic coder in the LAScompression algorithm is a byte stream therefore the arithmetic coder with byte alphabet and static input symbol model is used.

## 5. Hardware Architecture of LIDAR Data Compression

The main objective of developing hardware LIDAR data compression is to relieve the processor of the additional processing load introduced by software compression. Moreover it is also desirable that the compression doesn't reduce the throughput of the file transfer hence the speed of the hardware architecture was an important factor during its development.

**Figure 3.**
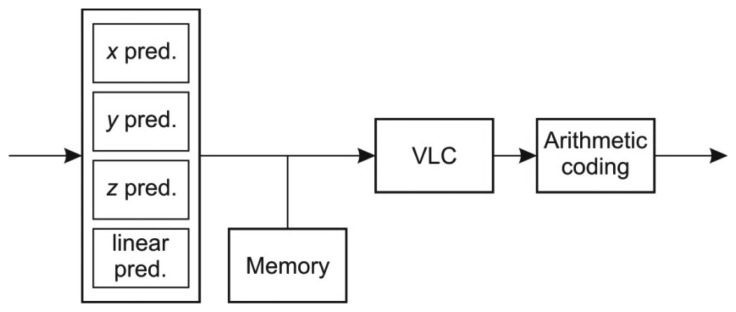
The structure of the hardware LIDAR data compressor.

The general architecture of the hardware compressor resembles the structure of the LIDAR compressor depicted in Figure 3. It consists of three main modules: predictive coder, variable length coder and arithmetic coder. While the variable length coder and the arithmetic coder are unified for all LIDAR record attributes the prediction coder depends on the type of the attribute.

The arithmetic coder defined by the LAScompression algorithm employs static probability model, hence a two-pass processing is required. This can be achieved in three ways:

1. Storing the original LIDAR data in the memory and performing two consecutive prediction and variable length encodings. The source model used for arithmetic coding is determined in the first pass, and the arithmetic coding is performed in the second pass.
2. Storing the results of the prediction in a memory for subsequent processing. In the first pass the variable length encoding is performed and the source model is determined. In the second pass the variable length encoding is repeated and the arithmetic coding is performed,
3. Storing the results of prediction and variable length encoding in a memory for subsequent arithmetic coding. The source model used for arithmetic coding is determined in the first pass, and the arithmetic coding is performed in the second pass.

The second method was chosen since it requires minimal storage resources and it enables parallel as well as sequential processing of the LIDAR data attributes.

The LIDAR data compression algorithm [12] processes LIDAR data in packets which allows the use of indexing data for easier searching. Each LIDAR data attribute could be processed in parallel as a separate data stream which would significantly increase the data throughput. However the LIDAR data are handled in a system via a data bus (*i.e.*, a PCI bus) which limits the overall data throughput. Thus it is not prudent to insist on fully parallel architecture and some modules, like arithmetic coder, can be reused for different data streams.

The set of fixed-length point data records of the LAS format represents the input data stream of the hardware LIDAR data compressor. The format of the point data record varies with versions of the LAS format, however all versions of LAS format share a common part [11]. While the whole point data record could be processed at once using highly parallel hardware architecture and achieving maximal data throughput, it would require a huge amount of hardware resources. To limit the hardware resource requirement while still enabling high data throughput, the data width of the hardware data compressor can be reduced to match the size of the largest point data attribute which is 32 bit.

### 5.1. X-Coordinate Prediction Coder

**Figure 4.**
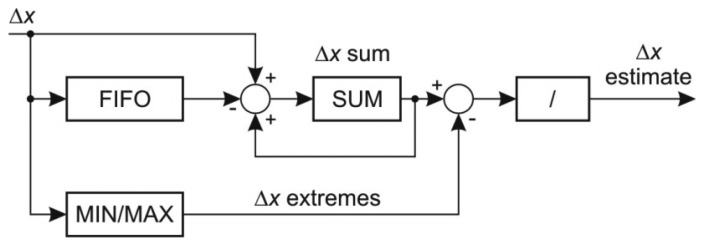
The structure of *x*-coordinate predictor.

The prediction of *x* coordinate is performed by averaging the last *k_x_* coordinate changes while omitting the extreme values. The prediction of the *x* coordinate change is subtracted from the actual coordinate change to form the error value at the output. The structure of the *x* coordinate prediction coder is shown in Figure 4.

In order to calculate the average last *k_x_* values, the sum of these values must be determined first. The sum is calculated by on-line adding the new value and subtracting the retired value which fell out of the averaging window. The retired value can be determined by delaying the input values by *k_x_* periods and is implemented by a FIFO structure. Extreme values are then subtracted from the total sum

**Figure 5.**
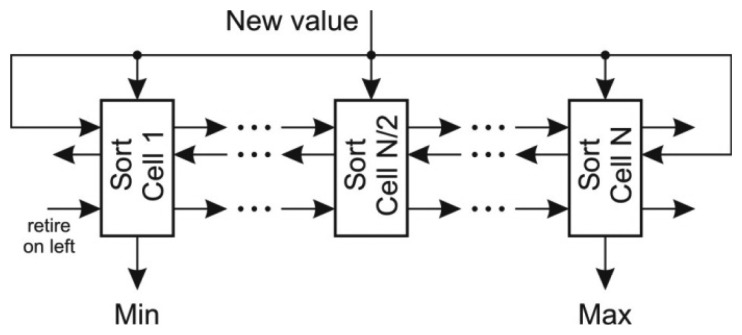
Insertion sort structure.

The most difficult task in the prediction of *x* coordinate is to determine the extreme coordinate changes. The straightforward approach to scan the FIFO requires *k_x_* iterations and is not suitable for online algorithm implementation. Better approach would be to use a priority queue for determining both minimal and maximal value, however the deletion of an arbitrary element from the priority queue is a demanding task. A sort structure with FIFO capabilities [25] proved to be an efficient solution in extracting the extreme values. This structure, depicted in Figure 5, implements an insertion sort where each value is stored within a sort cell together with its retire count. The retire count mimics the FIFO behavior. When the retire count of individual cell drops to zero, the cell content is overwritten by the new value and the retire count is reset. The new value is compared with each cell in order to determine its position. With respect to the position of the retiring cell there are three possibilities:

- The retiring cell is on the left of the new value position: the cell values between retiring cell and the new value position are shifted left,
- The retiring cell is on the right of the new value position: the cell values between the location of new value and retiring cell are shifted right,
- The retiring cell is at the location of the new value: the new value overwrites the retiring cell and its retire count is reset.

Note that the retire count must be shifted alongside with the value of the cell. The given structure maintains the sorted array in a single clock period. The extreme values are acquired at the output of the first and the last cell of the sort array with FIFO capabilities. They are subtracted from the current sum of the last *k_x_* values.

To calculate the average the sum has to be divided by the number of the summed values. While the division operation is difficult to implement and it may require several clock cycles, the result can be obtained by multiplication with the inverse of number of elements in FIFO. Since the denominator changes only until the FIFO buffer is full and its size *k_x_* is relatively low, there are only a few different denominators and their inverses can be pre-stored.

### 5.2. Y-Coordinate Prediction Coder

The prediction of *y* coordinate is more demanding than the prediction of *x* coordinate since it consists of two prediction rules:

- Finding the latest *x* coordinates change (∆*x*) that is close to the current ∆*x* in last *k_y_* records, and using the corresponding *y* coordinate change (∆*y*) as an approximation for the current ∆*y*,
- Linear prediction based on the last coordinate changes.

Like in the *x* coordinate prediction coder, a FIFO structure is used to store the last *k_y_* of *x* and *y* coordinates changes (∆*x*, ∆*y*). The latest record whose ∆*x* is close enough to the current ∆*x*, could be determined by scanning over the ∆*x* FIFO array. However, such a search is too slow especially since the depth of the FIFO array is quite large. The alternative is to use a modified FIFO structure with a built-in comparison of each stored ∆*x* value with the current ∆*x* denoted as FIFO search array. Such an altered FIFO structure returns a flag for each FIFO cell indicating that a cell ∆*x* value is close enough to the current ∆*x*. The position of the latest cell with acceptable value within the FIFO structure is determined by counting the most recent non-acceptable flags using Leading Zero Count structure (LZC). This can be achieved in a single clock period and the address is used to look-up for the corresponding ∆*y* in the FIFO structure holding *y* coordinate changes.

In the case when there is no similar ∆*x* in the FIFO search array, a linear predictor is used. Linear predictor uses only the last two coordinate changes and requires only two registers to store them. On the other hand, the linear predictor employs a division which by itself is a demanding operation. While the non-restoring division can be implemented to perform a division in a single cycle, such implementation has a substantial delay and reduces the maximum clock frequency and hence the data throughput. To maximize the data throughput, a non-restoring division was developed using a pipelined architecture where a division is performed in several stages. Single division requires several clock cycles. However, when multiple divisions are performed, the implemented pipeline structure allows for parallel execution. On the other hand, the pipelined architecture introduces a latency of the divider. A delay circuit was applied to synchronize the operations of all modules.

**Figure 6.**
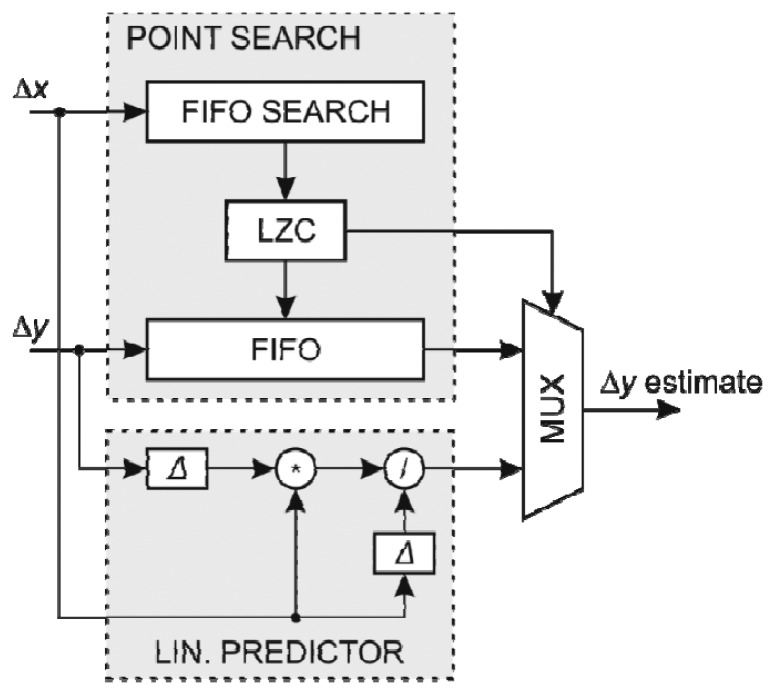
The structure of *y*-coordinate predictor.

The structure of the *y* coordinate predictor using FIFO search array with the search feature is shown in Figure 6. The prediction of the *y* coordinate change is subtracted from the actual coordinate change to form the error value at the output of the coder.

### 5.3. Z-Coordinate Prediction Coder

The prediction of the *z* coordinate resembles the prediction of the *y* coordinate but with added complexity. It requires two search arrays with FIFO capability for the *x* and *y* coordinates changes, respectively, and its linear predictor consists of two linear predictors depending on the *x* coordinate and *y* coordinate changes with the prediction selector based on the recorded accuracy of both predictors. Since *k_y_* and *k_z_* are equal, the FIFO search array for the *x* coordinate changes is identical to the structure used in the *y* coordinate prediction coder, so it can be reused. An additional FIFO search array is required for searching through the last *k_z_y* coordinate changes. This array could be reused as a FIFO structure in ∆*y* estimation. On the other hand, a preferred solution is to use a separate FIFO array which leads to higher clock frequency and occupies less FPGA resources.

When both the *x* and *y* coordinate changes are within the predefined margins, the corresponding *z* coordinate change is used as an approximation. Otherwise a prediction using linear predictor based on the coordinate, which gave better estimations in the last *k_z_* predictions, is used.

**Figure 7.**
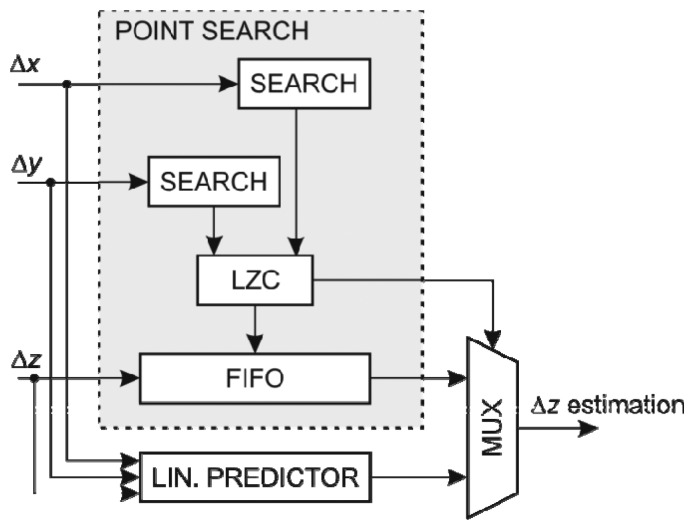
The structure of *z*-coordinate predictor.

The structure of the *y* coordinate predictor using FIFO search arrays is shown in Figure 7. Since there are separate linear predictors using the *x* and *y* coordinate, respectively, two dividers are required. However the divisor of the predictor using *x* coordinate is the same as the divisor used in the linear predictor of the *y* coordinate thus the divisor can be adapted to operate on two dividends simultaneously. In addition there is a selector circuit for choosing the most suitable linear approximation.

### 5.4. Other Attributes Prediction Coder

The prediction of other attributes of a LIDAR point is determined by a linear or constant predictor depending on the behavior of the attribute. While the constant prediction coder uses the last value as an approximation the linear predictor uses the last attribute change for the estimation of the following attribute change. Both predictors are very simple and use a single register.

### 5.5. Variable Length Coder

**Figure 8.**
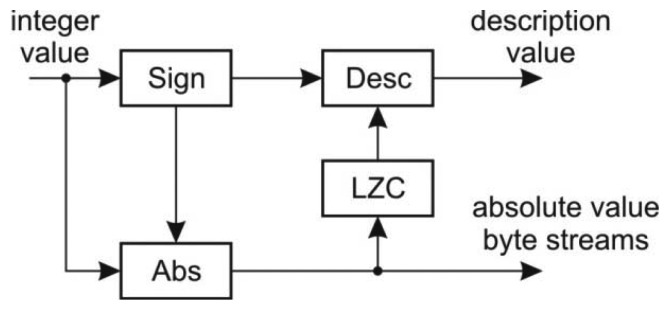
Variable length coder.

The variable length coder encodes the 32-bit integer values into a description stream and four byte streams corresponding to a particular byte within the word. The structure of the coder is shown in Figure 8.

At the input there is a sign detector which is used for the description value calculation as well as for the calculation of the absolute value. The modified LZC module determines the number of non-zero bytes within the word value in order to determine the description value.

### 5.6. Arithmetic Coder

**Figure 9.**
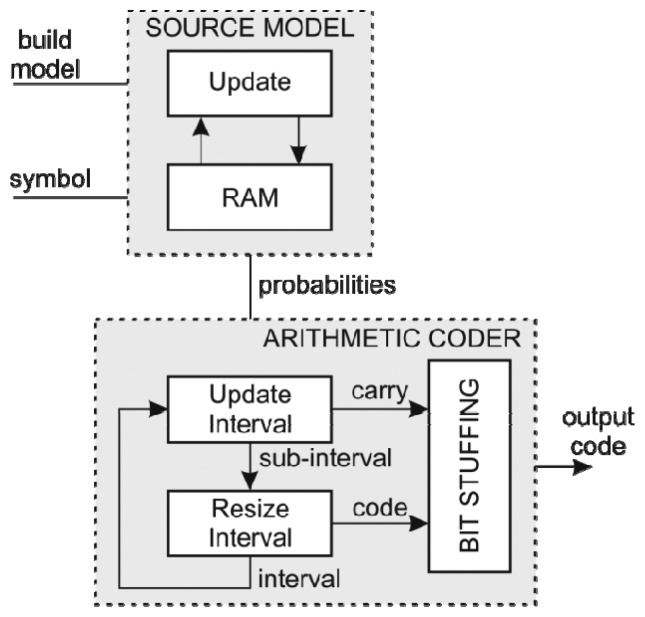
Hardware arithmetic coder.

The hardware arithmetic coder uses a static 256-symbol model [26]. Using a byte symbol model enables the processing of a complete byte at once and at the same time achieves better compression. The hardware structure of both the source model and the arithmetic coder is depicted in Figure 9.

Source model includes a small RAM with additional logic required to determine the cumulative probabilities of each symbol in the first pass. Since the number of the input symbols is arbitrary, a divider is needed to calculate the probabilities. In the second pass the source model behaves as a plain RAM device.

The Rescale and Bit-Stuff modules of the arithmetic coder [26] were modified to use barrel shifters in order to perform rescale during single clock period. Originally at each rescale iteration only a single bit was shifted out of the Rescale module which limited the overall data throughput. Using barrel shifters, the rescale is done in parallel with the update in a single clock period. This in turn results in eight times higher data throughput. On the other hand this implementation requires more hardware resources and longer critical paths, which reduce the maximum clock frequency.

## 6. Hardware Implementation of LIDAR Data Compression

The LIDAR data compressor was implemented on a Field Programmable Gate Array (FPGA)-based platform manufactured by Xilinx Inc. (San Jose, CA, USA). FPGA is an integrated circuit composed of an array of logic blocks connected through interconnection switches. Both logic blocks and interconnections can be configured by the user using the dedicated equipment in order to implement the required logic functionality. Contemporary FPGA devices contain a substantial number of logic blocks and RAMs and can implement complex digital designs like multiprocessor systems and even networks-on-chip. Compared to traditional integrated circuits like microprocessors or application-specific integrated circuits (ASICs) their functionality can be tailored for a specific application. While their costs are higher than the costs of microprocessors, they are especially suitable for prototyping and they are cost effective in the case of low-volume production.

**Figure 10.**
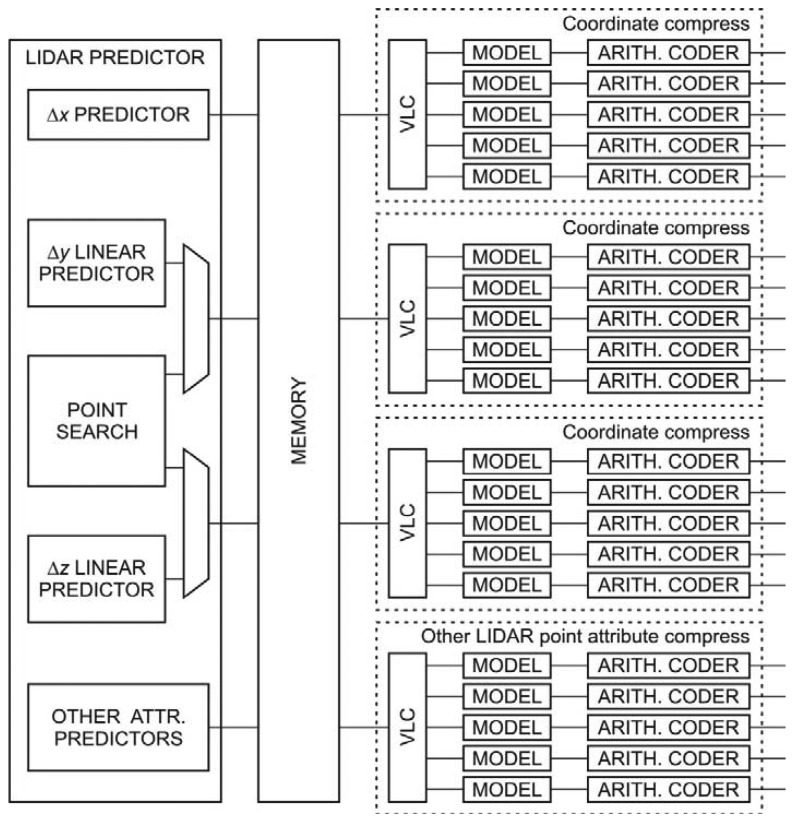
Parallel architecture of LIDAR compressor.

The hardware LIDAR data compressor must be implemented on a platform with suitable communication interface in order to take advantage of its speed. A PCI interface is a suitable interface for implementation of an acceleration device. The latest Xilinx FPGA families like Virtex5, Virtrex6, and Spartan6 support such high speed interfaces. The chosen platform for hardware LIDAR compressor was Xilinx XUPV5 development board, which is equipped with PCI-E interface and is populated with a mid-range Virtex-5 XC5VLX110T FPGA device. The hardware LIDAR data compressor was synthesized using Xilinx ISE 14.2 development environment. The fully parallel structure of the LIDAR compressor is shown in Figure 10.

Initially all individual modules were synthesized separately. The resource utilization and the corresponding maximal clock frequency are given in Table 1. While some modules of LIDAR compressor are relatively simple and do not require a lot of hardware resources, others, like *y* coordinate predictor, occupy significant portion of the target FPGA device.

As can be seen in Table 1, 50 MHz clock frequency can be applied to the developed modules. The data throughput can be determined considering the width of the data bus and the number of clock periods (in our case: 2) required for the data processing.

A fully parallel structure yields the highest data throughput since the whole data of a LIDAR point is processed within two clock periods: one clock period for building the source model, and another for the arithmetic encoding. In this case, the width of the data bus is 160 bits. The data throughput is slightly below 4 Gb/s which is in the range of the data throughput of PCI-E 2.0 as well as SATA 3.0 interface. In the fully parallel architecture the communication interface might present a bottleneck. Such architecture also consumes a lot of FPGA resources and might not fit onto a mid-range Virtex5 devices.

An architecture, where a single LIDAR point coordinate is compressed at once presents a good trade-off between the processing speed and the size of the design. Since the size of the LIDAR data record is five times bigger than the size of a single coordinate the data throughput is 5 times smaller. Such architecture also easily fits into the mid-range Virtex5 device leaving non-occupied resources for PCI interface implementation.

The data throughput of the hardware LIDAR compressor was compared with software compression algorithm implemented in C++ on a Linux operating system with a 2.40 GHz Intel Core2 Quad Q6600 processor and 8 GB RAM. The comparison results for compression of the 840,000 LIDAR data points and are shown in Table 2.

In the case that smaller FPGA devices were used and the LIDAR data compressor along with PCI interface doesn't fit in the device, some tradeoffs among the data throughput and resource utilization must be considered. The coordinate predictor should be redesigned to use FIFO scans in order to reduce the design size. However such design would be significantly slower.

## 7. Conclusions

Developed hardware LIDAR compressor is fully compatible with the existing software compression algorithm. Consequently, the two alternatives are interchangeable in practice. In hardware compression, the user can chose among the implementations with different levels of parallelism which, on the other hand, require different amount of hardware resources and impact the selection of the target FPGA device. In a fully parallel version, the hardware compression is about 250 times faster than software processing. The compression of the LIDAR data is implemented on a FPGA development platform thus it is primarily applicable to data servers used to store the acquired LIDAR data. While the above solution may be regarded as an important step in speeding up LIDAR data compression, an additional improvement can be obtained by a modification of LAS algorithm, in which arithmetic coding with adaptive source model is used. Development of the adaptive source model with high data throughput remains subject of our current research.

## Figures and Tables

**Table 1.** FPGA resources utilization for LIDAR compressor modules.

**Module**	**Slice FF**	**LUTs**	**BRAMs**	**DSP48 s**	**Max. Clock [MHz]**
X predictor	348	1,042	0	4	58.1
Y linear predictor	713	2,357	0	4	117.5
Z linear predictor	1,416	4,995	0	8	92.2
Point search	6,400	13,871	0	0	130
**LIDAR predictor**	**9,264**	**29,864**	**0**	**16**	**55.3**
VLC	0	81	0	0	-
Source model	856	2,338	1	0	117.5
Arithmetic coder	153	531	0	2	86.7
**Coordinate compress**	**4,031**	**11,586**	**4**	**8**	**82.0**

**Table 2.** Comparison of data throughput of different implementations.

	**Fully Parallel**	**Coordinate Compress**	**Software**
Time [s]	0.0336	0.168	8.1
Throughput [Mb/s]	3,999	799	16.6
